# High-Precision Measurement of Microscales Based on Optoelectronics and Image Integration Method

**DOI:** 10.3390/mi15091162

**Published:** 2024-09-17

**Authors:** Yanlong Zhu, Yinbao Cheng, Hongtang Gao, Shuanghua Sun, Xudong Zhang, Liang Xue, Jiangwen Tang, Yingqi Tang

**Affiliations:** 1 College of Metrology Measurement and Instrument, China Jiliang University, Hangzhou 310018, China; p22020854189@cjlu.edu.cn (Y.Z.); cyb@cjlu.edu.cn (Y.C.); 2 Division of Dimensional Metrology, National Institute of Metrology, Beijing 100029, China; sunshh@nim.ac.cn (S.S.); zhxd@nim.ac.cn (X.Z.); 3 National Institute of Measurement and Testing Technology, Chengdu 610021, China; xueliang@nimtt.com (L.X.); tangjiangwen@nimtt.com (J.T.); 4 School of Optics and Photonics, Beijing Institute of Technology, Beijing 100081, China; 7520230008@bit.edu.cn

**Keywords:** micro and nanometrology, microscale, photoelectric microscope, integrated line detection, laser interferometer, image processing

## Abstract

Currently, there are various types of microscales and the conventional line detection system usually has only one detection method, which is difficult to adapt to the diverse calibration needs of microscales. This article investigates the high-precision measurement method of a microscale based on optoelectronics and the image integration method to solve the diversified calibration needs of microscales. The automatic measurement and processing system integrates two methods: the photoelectric signal measurement method and the photoelectric image measurement method. This article studies the smooth motion method based on ordinary linear guides, investigates the method of reducing the cosine error of a small-range interference length measurement, proposes an image-based line positioning method, and studies the edge and center recognition algorithms of the line. According to the experimental data, the system’s measurement accuracy was analyzed using the photoelectric signal measurement method to measure the 1 mm microscale, the maximum difference from the reference value was 0.105 μm, the standard uncertainty was 0.068 μm, and the absolute value of normalized error was less than 1. The accuracy of the image measurement method to measure the 1 mm microscale was consistent with that of the photoelectric signal method. The results show good consistency in the measurement results between the two methods of the integrated measurement system. The photoelectric signal method has the technical characteristics of high measurement efficiency and high accuracy, while the pixel-based measurement of the image method has two-dimensional measurement characteristics, which can realize measurements that cannot be realized by the photoelectric signal method; therefore, the measurement system of optoelectronics and image integration is characterized by high precision and a wide range of measurement adaptations.

## 1. Introduction

As a special kind of line scale, the microscale is usually used to calibrate the indication, magnification, and other technical parameters of optical microscopic equipment with measurement function, which are widely used in the fields of life sciences, machine vision, microelectronics, etc. Therefore, the calibration of microscales is crucial. With the rapid development of many related fields, the types of microscales are gradually increasing, and it is necessary to study the microscale measurement system that integrates multiple methods to meet the calibration needs of multiple types of microscales.

At present, the measurement method of the microscale line is divided into two kinds, which are the photoelectric signal measurement method and the image measurement method [1], and both methods use microscopes to detect the line. The photoelectric signal measurement method converts the line’s image into an electric signal through the slit and the optoelectronic converter device, and determines the center of the line through the processing of the electric signal. Wang Li et al. provided a detailed introduction to a new multifunctional 1 m laser interference length comparator, which uses a dual slit photoelectric microscope for line positioning. Using this length comparator to measure a first-order glass line scale, the expanded uncertainty is *U* = (0.1 + 0.5*L*) μm, *k* = 3 [2]. Liu Tingting et al. studied a 2 m laser interference length comparator, which uses a dual tube differential dynamic photoelectric microscope for line positioning [3]; NIM’s GAO Hongtang et al. studied a dynamic measurement method for a grating pitch based on a photoelectric microscope, using this method to measure a grating with a length of 1 mm and a pitch of 10 μm; the expanded uncertainty is less than 0.1 μm [4]. Lu Yutong and others have developed a two-dimensional dynamic photoelectric microscope, which can simultaneously detect lines in two vertical directions. In each direction, the double slit photoelectric detection method is used, and the positioning accuracy of the system in the x and y directions is better than 0.1 μm [5].

The image measurement method captures images of microscales using calibrated cameras and calculates parameters using image processing algorithms. Pavol Kajánek et al. developed a length comparator assembled from low-cost components, calibrated using laser interferometers, line scales, and image acquisition and processing programs. During the process, an application was developed to automatically manage the calibration process, and the universality of the comparator and application program was verified [6]. Tan Dongxing et al. developed a line scale detection device based on technologies such as air floating guide rails, laser length measurement, and image positioning. The device was used to measure second-class standard metal line rulers, and the expanded uncertainty of the measurement results was *U* = (0.2 + 0.8*L*) μm, *k* = 3 [7]. Li Qiaoli et al. studied a line scale detection system based on CCD vision, marble guide rails, and laser interferometers, with a focus on the line center extraction algorithm. The system was used to measure a 1 m third-order standard metal line ruler with an extended uncertainty of 9 μm and *k* = 2 [8]. Ma Jianqiu et al. proposed a microstructural linewidth measurement method based on translational difference. Three microscopic images were collected by a two-step translational difference, and then subtracted to obtain two differential images. The linewidth measurement was converted into a differential pulse distance measurement, and the relative uncertainty of this method for measuring a 30 μm linewidth is 37% (*k* = 1) [9].

The above research results show that the photoelectric signal measurement method and image measurement method have their own characteristics; the photoelectric signal measurement method has a higher measurement accuracy, but due to the single function, the image measurement method is currently applied more, and due to the large number of the types of microscales, the single measurement method cannot meet the needs for the calibration of multiple types of microscales. So, in this paper, we designed and built an optoelectronic and image integrated microscale measurement system, which integrates optoelectronic signal measurement and optoelectronic image measurement to increase the measurement function. This paper will analyze the design principles of the measurement system, discuss in detail the key methods used, demonstrate the system’s measurement effectiveness, and evaluate the system’s measurement uncertainty. Finally, the system studied in this article is compared with existing measurement systems to demonstrate its measurement accuracy.

## 2. Measurement Principles and System Composition

**Figure 1:**
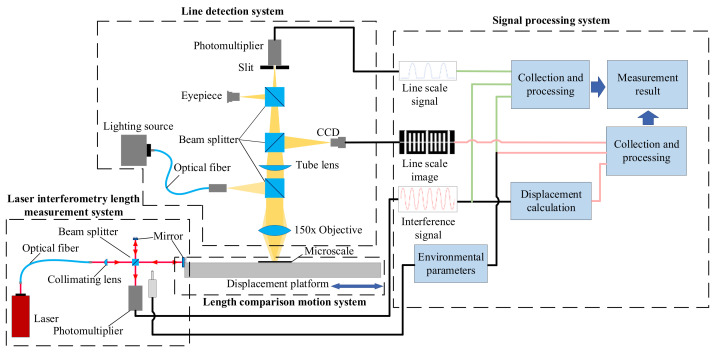
System schematic diagram.

The principle of the optoelectronic and image integrated microscale measurement system is shown in Figure 1:

The system incorporates the photoelectric signal measurement method and the image measurement method. The system is divided into the motion system, laser interference length measurement system, integrated line detection system, and signal processing system. The motion system is an important foundation of the whole measurement system, which is interconnected with the laser interferometry system and the scribe detection system. The motion system consists of a linear guide, slider, and drive motor, which can drive the microscale to carry out smooth motion in both directions automatically. The laser interferometric length measurement system is based on the principle of Michelson interference; due to the high heat generation of the laser, it has a significant impact on the temperature near the measurement optical path. Therefore, a separate design between the laser and the interference optical path was adopted, and the laser is conducted into the interferometric optical path with an optical fiber; the laser interferometric optical path adopts a planar reflector, which can effectively reduce the size of the interferometric optical path and the influence of the environment near the interferometric optical path, which facilitates integration. A photomultiplier was used for photoelectric conversion, and a single channel interference signal was the output. The integrated line detection system is based on the principle of infinity-corrected optical imaging, and adopts a reflective illumination method, in which the illumination light is conducted into the imaging system through an optical fiber where the objective and the tube lens image the line, and the image of the line is formed into the eyepiece observation surface, the slit surface, and the charge-coupled device (CCD) imaging surface through the beam splitter, respectively, so that observation and measurement can be carried out. A suitable slit is selected according to the width of the line image, and the image can be measured when the microscale moves slowly with the motion system; the photomultiplier after the slit will scan the line signal, the integrated line detection system can be used for line positioning and measurement through the single slit photoelectric signal method, or through the image method. After replacing the eyepiece with a slit, the line positioning and measurement can be carried out through the double slit photoelectric signal method and users can choose different methods according to the actual situation of the signal processing system. The system is adaptable. The signal processing system used in this article is a software processing system. The signal processing system collects line signals, laser interference signals, line images, environmental data, etc., with the computer, and processes and calculates them through the computer software system to obtain the final measurement results.

## 3. Key Technologies and Methods

### 3.1. Smooth Motion Method

**Figure 2:**
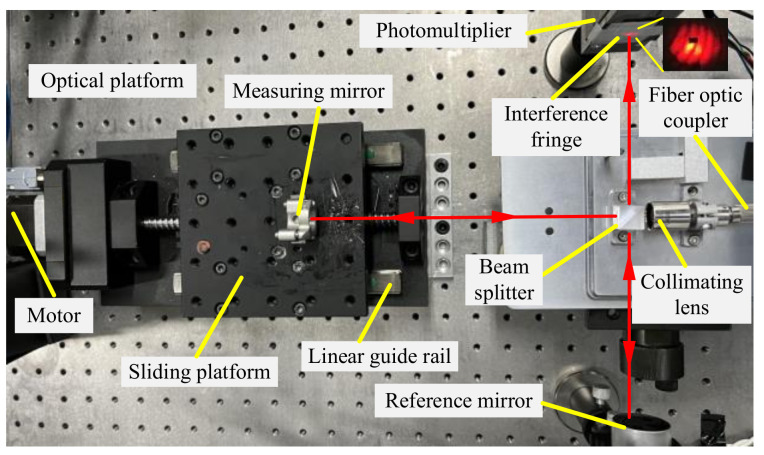
Experiments on transmission performance testing of motor drive methods.

**Figure 3:**
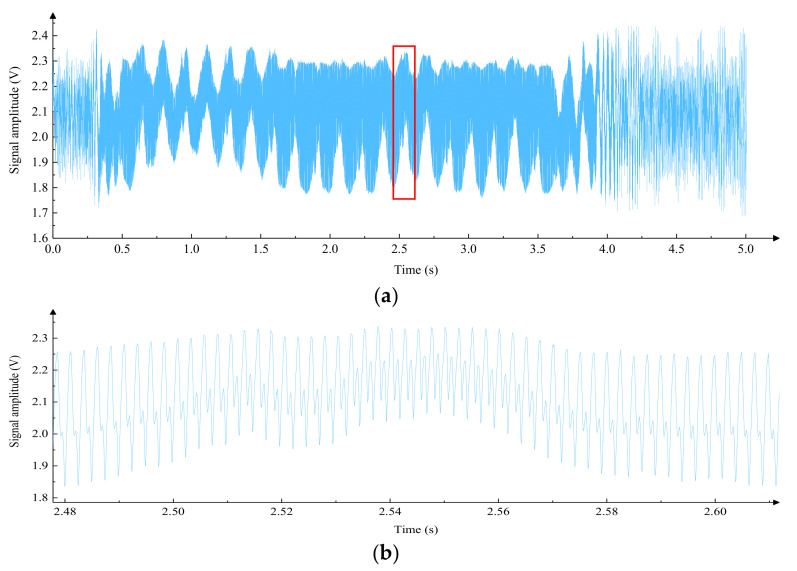
Laser interference signals for stepper motor drive methods. (**a**) Interference signals in the stepper motor drive mode within 5 s; (**b**) local magnification of the interference signal (The red box area in Figure 3a) in the stepper motor drive mode over a period of 5 s.

When carrying out laser interference for length measurement, the smoothness of the motion is required to be high, and the article is based on an ordinary linear guide to study the method of smooth motion. At present, the drive of precision instruments is mostly driven by stepper motors and servo motors. To compare the applicability of the two driving methods in this system, a linear guide rail was selected, and different driving methods were used. The stability was judged by observing the laser interference signal, as shown in Figure 2; by using an elastic coupling, the motor shaft was directly connected to the transmission screw concentrically, avoiding radial force on the transmission screw. The movement direction of the linear guide was adjusted to be parallel to the laser interference optical axis, and the slider was driven by the motor to move on the linear guide, collecting interference signals for analysis. The experimental results of the stepper motor drive method are shown in Figure 3, where Figure 3a shows the interference signal collected in 5 s, and Figure 3b shows the local amplification of Figure 3a. The drive circuit used in the experiment is 1/64 on the fine fraction of the input control signal, the frequency of the control signal is 400 Hz, and the speed of the slider is about 0.14 mm/s.

**Figure 4:**
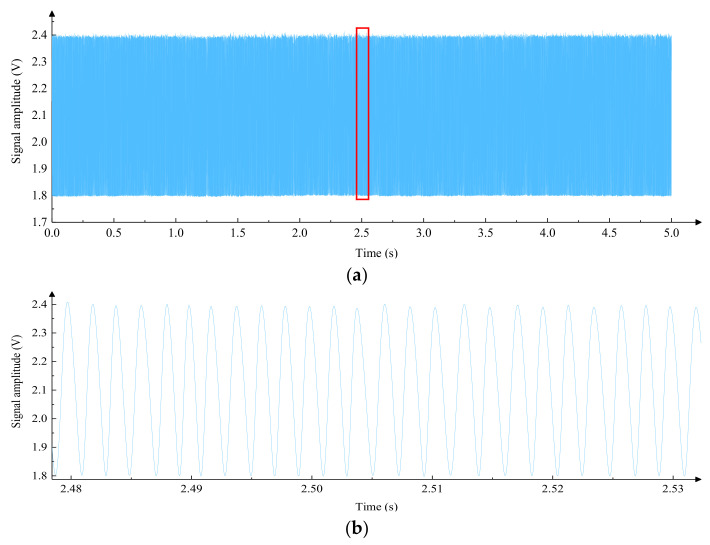
Laser interference signals for servo motor drive methods. (**a**) Interference signals in the servo motor drive mode within 5 s. (**b**) Local magnification of the interference signal (The red box area in Figure 4a) in the servo motor drive mode over a period of 5 s.

By analyzing Figure 3a, it can be seen that the low-frequency fluctuation of the interference signal is significant. After local amplification, it can be observed that there was a phase distortion in the interference signal in Figure 3b, which will affect measurement accuracy and even cause errors. The stepper motor was replaced by a servo motor for driving, and the laser interference experiment was carried out again; the speed of the slider was about 0.15 mm/s, and the collected interference signals are shown in Figure 4 where Figure 4a shows the interference signal in 5 s, and Figure 4b shows the local amplification of Figure 4a. It can be seen that the interference signal of the servo motor driving method has a uniform amplitude and a stable period, which indicates that the smoothness of motion is better.

For the same linear guide, by using different driving methods, the smoothness of movement is different; driven by a servo motor, the interference signal is smooth and uniform, which is conducive to improve the measurement accuracy. Therefore, the integrated measurement system adopts servo motor drive mode.

### 3.2. Miniature Laser Interferometer

In this study, a miniature laser interferometer was built. The length measurement system was based on the Michelson interference principle, and due to the large size of the angular cone prism, it was difficult to manufacture and install, which was not conducive to the miniaturization and integration of precision instruments. In order to reduce the size of the laser interferometer, improve the efficiency of the optical path adjustment, and reduce the influence of the environment on the measurement optical path, this measurement system separates the laser from the interferometric measurement optical path and uses multimode fibers for laser transmission. In the interferometric measurement optical path, a planar mirror was used as the reference mirror (M1) and the measurement mirror (M2), which improves the efficiency of optical path adjustment and has high measurement accuracy.

**Figure 5:**
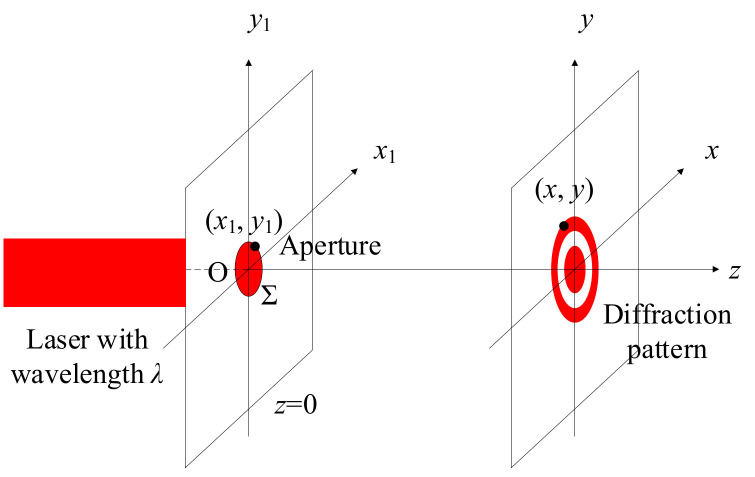
Diffraction model of circular aperture.

Usually, the heat generation of the laser is high, and the proximity to the measurement optical path will lead to a significant increase in the ambient temperature, which also affects the measurement accuracy. In order to reduce the impact of laser heat generation on the measurement optical path, the light source and the measurement optical path were designed as a separate unit, the laser light was guided to the measurement optical path through the optical fiber, the laser light was transferred to the measurement optical path through the optical fiber, and the single-mode optical fiber was used to transmit the laser light because of the lower coupling efficiency and the difficulty of adjustment; therefore, the multimode fiber was used for laser transmission. An aperture was used to limit the beam diameter in the interferometric optical path, and the diameter of the aperture was determined according to the diffraction principle and the allowable error of the system. The small-hole diffraction model of the Gaussian beam is shown in Figure 5, in which ***∑*** is the surface domain of the aperture, the center of the aperture O is the origin of the coordinates, (*x*_1_,*y*_1_) is the point on ∑, and (*x*, *y*) is the point on the diffraction pattern.

According to the model deduction, the intensity distribution on the diffraction pattern is as follows [10]:

(1) $$I\left(x,y,z\right)={\left|\frac{1}{\lambda iz}e^{\frac{2\pi i}{\lambda }z}\iint_{\Sigma }E\left(x_{1},y_{1}\right)e^{\pi i\frac{x_{1}^{2}+y_{1}^{2}-2x_{1}x-2y_{1}y}{\lambda z}}dx_{1}dy_{1}\right|}^{2}$$

In the formula, $I\left(x,y,z\right)$ is the light intensity distribution on the diffraction pattern, *λ* is the wavelength of the laser, and *E* (*x*_1_, *y*_1_) is the electric field distribution on the aperture plane, which can be calculated according to the electric field distribution of Gaussian beams.

**Figure 6:**
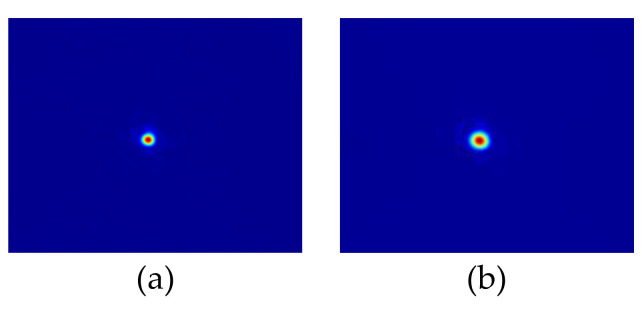
Laser spot at the near and far ends of the laser interferometer. (**a**) Laser spot at the near ends of the laser interferometer. (**b**) Laser spot at the far ends of the laser interferometer.

In order to ensure the consistency of the incident light direction of the interferometric optical path with the direction of the measurement axis, the measurement mirror in the interferometric optical path was replaced with a camera, the slider was moved to both ends of the linear guide, and two images were taken by the camera to observe whether the center of the beam in the two images coincided. After adjustment, the images are shown in Figure 6a,b, the coordinates of the centers of the beams are (609, 529) and (607, 532), the pixel size is 5.2 μm × 5.2 μm, the full travel length of the guide is 55 mm, the deflection angle between the whole interferometric optical path and the measurement axis is 0.019°, and the measurement length error for every 1 mm movement is 0.05 nm.

**Figure 7:**
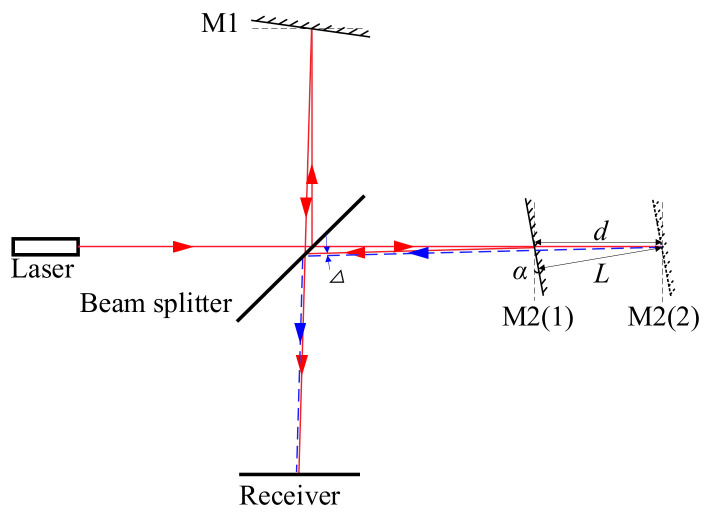
Cosine error of interference optical path.

In an ideal laser interference optical path, M1 and M2 should maintain a strict vertical relationship, but in an actual optical path adjustment, interference fringes may also appear in nonvertical situations [11], as shown in Figure 7. In this optical path state, interference fringes will appear. When the measurement length is very short, the deviation of the optical axis Δ is extremely small, and the interference fringes will continue to exist. At this time, if M2 moves from position 1(M2(1)) to position 2(M2(2)), the blue arrow in Figure 7 represents the laser reflection direction of M2 moving to position 2 (M2 (2)), the distance of movement is *d*, but the distance measured by the interferometer is as follows:

(2) $$L=d\times \cos\left(\alpha \right)$$

The difference between the actual movement distance and the distance measured by the interferometer is called the cosine error [12]. If the cosine error is ΔL, the following applies:

(3) $$\Delta L=L\times \left(\frac{1}{\cos(\alpha )}-1\right)$$

In precision measurement, where *α* is usually very small, Equation (3) can be simplified to the following:

(4) $$\Delta L=\frac{L\times \alpha^{2}}{2}$$

**Figure 8:**
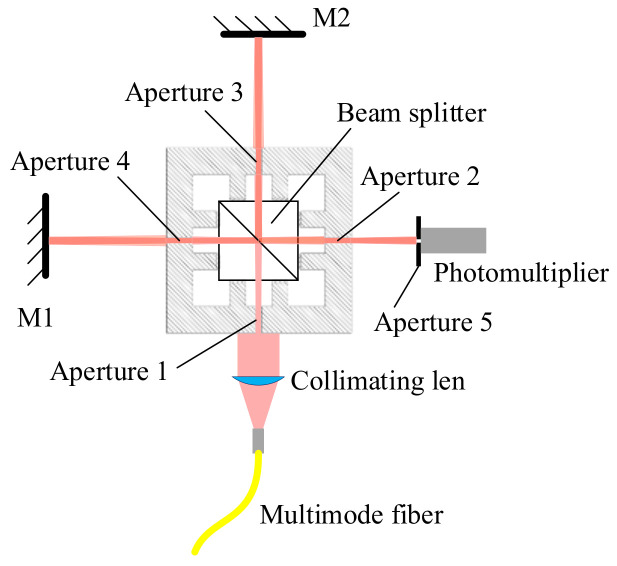
Structure of laser interferometer optical path for cosine error reduction.

The calculation shows that when *L* is 1 mm, the deflection angle is 0.5°, and the cosine error reaches 38nm, in small length measurements, the influence of cosine error on measurement results cannot be ignored, so it is necessary to study methods for reducing cosine error in small length measurements. The interference optical path structure is shown in Figure 8; there are four apertures of the same height around the beam splitter and the distance between the two opposite directions of the apertures is 25 mm. A laser passing through a collimating lens undergoes diffraction every time it passes through an aperture, and each aperture only allows zero-order diffracted light to pass through, assuming that the diameter of the apertures is 0.2 mm. When the diffracted light emitted from one small hole reaches the next small hole, the diameter of the zero-order diffracted light is 0.19 mm, and then they are a part of the first-order diffracted light in the next aperture. In order to avoid the influence of the diffraction pattern on the interference, the diameter of the aperture needs to be less than 0.2 mm. If the diameter of the aperture is 0.1 mm, at this time, the diameter of the zero-order diffracted light in the next aperture is 0.39 mm. Due to the limitation of small holes, the optical power attenuation of the laser during propagation is significant. Therefore, a photomultiplier tube is used to detect interference signals, and the application of multimode fibers can also increase the power of the incident light. After adjustment, the optical path deviation should not exceed 0.1 mm. When the length of the measuring arm is greater than 60 mm, the cosine error caused by the mirror deviation can be controlled within 2 nm.

### 3.3. Integrated Line Positioning System

The line positioning system is one of the keys of the microscale measurement system and its core is to find the center position of the line [13,14]; for different microscales, different line positioning methods need to be used according to the shape of the line. The conventional line positioning system has a single function, which makes it difficult to calibrate some microscopic scales and increases the calibration costs. Therefore, this section describes the integration of the line positioning methods. At present, there are two widely used methods: the line positioning method based on photoelectric signals and the image-based line positioning method; both methods have their own characteristics. The line positioning method based on photoelectric signals can achieve the high-precision positioning of one-dimensional microscales, thereby achieving high-precision distance measurement. This method has high measurement efficiency but cannot locate the lines of irregular microscales and two-dimensional microscales. The contrast of some microscale lines is low, and it may be difficult to collect effective line signals; the image-based line positioning method has a wide range of applications and is not limited by the shape of the line. With the help of image processing technology, the discrimination ability can be improved. However, it is constrained by factors such as imaging distortion, and distortion is severe when it is far from the center of the image, which has a significant impact on the measurement accuracy of geometric parameters. This article is based on an infinite distance correction optical imaging system and combines the advantages of two methods.

**Figure 9:**
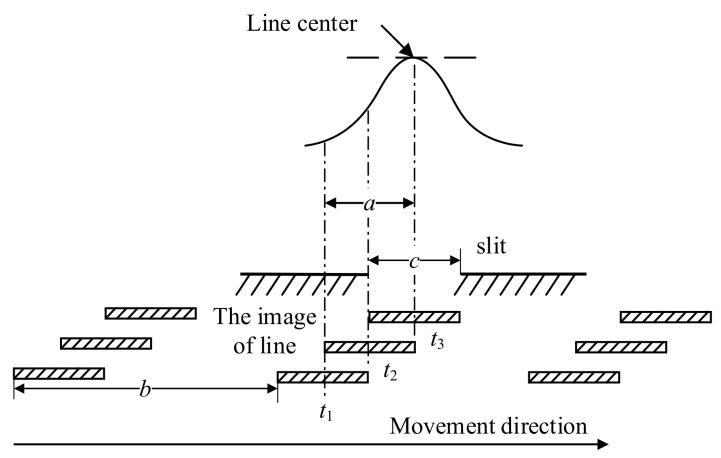
The principle of the line positioning method based on single slit photoelectric signals.

The principle of the line positioning method based on single slit photoelectric signals is shown in Figure 9, assuming the image width of the line is *a*, the distance between the images of two adjacent lines is *b*, and the slit width is *c*. In an ideal state, *a* is equal to *c*, and the line is magnified and imaged to the slit surface through optical amplification. When the image of the line moves at a uniform speed, the light flux entering the photomultiplier through the slit will show a regular change. By analyzing the optical imaging principle and the output signal of the photomultiplier, line positioning can be completed. When the center of the line coincides with the center of the slit, it is in the positioning state, and the photoelectric signal shows a peak position. According to the brightness of the line relative to the background light, the photoelectric signal is divided into an upper convex type and a lower concave type. When the image of the line is a bright stripe, the photoelectric signal is an upper convex type. When the image has dark stripes, the photoelectric signal is concave. In fact, there is a difference between *a* and *c*, and the complete line signal can be obtained by meeting the following conditions:

(5) b≥a+c

**Figure 10:**
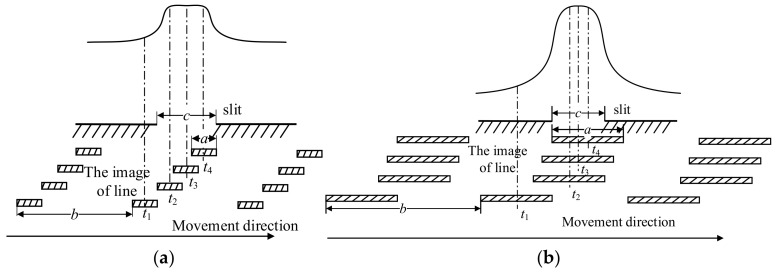
Principle of line signal generation in practical system. (**a**) Photoelectric signal at *a* < *c*. (**b**) Photoelectric signal at *a* > *c*.

The principle of line signal generation in the actual system is shown in Figure 10. Figure 10a shows the principle of line signal generation when *a* < *c*, and Figure 10b shows the principle of line signal generation when *a* > *c*. It can be found that the actual line signal is a flat top shape, and using software to process the signal has more advantages. Collecting the signal to the computer for calculation and processing can quickly determine the position of the line center.

**Figure 11:**
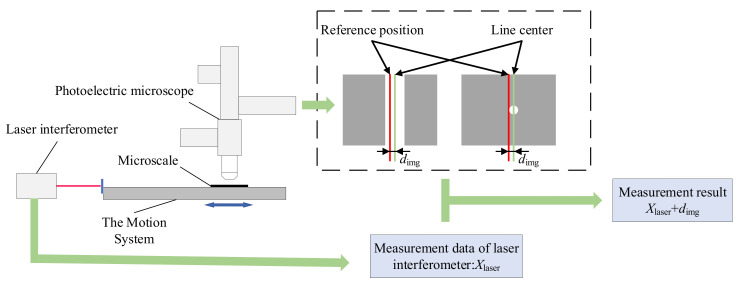
Principle of image-based line positioning method.

The principle of the image-based line positioning method is shown in Figure 11. This method is a static positioning method that uses the center of the image field of view as the reference position to reduce the impact of image distortion on the accuracy of line center positioning. The reference position in the image is used as the positioning line, and a 150× high magnification object is used. We moved the measured line to the image reference position, read the laser interferometer data, captured the line image, and calculated the pixel distance between the line center and the image reference position through image processing algorithms. The calibrated camera can convert the pixel distance into the actual distance. The indication of the laser interferometer and the calculated distance in the image were vector added to obtain the absolute position of the center of the line. The difference between the absolute positions of the two lines is the line spacing.

For any line, assuming that the indication of the laser interferometer is *X*_laser_, and the distance between the center of the line and the image reference position is calculated as *d*_img_ through image processing algorithms, the absolute position of the line is shown in Equation (6). We calculated the absolute position of each line, and the difference between the absolute positions of the two lines is the line spacing.

(6) $$X=X_{\mathrm{laser}}+d_{\mathrm{img}}$$

### 3.4. Data Processing Methods

#### 3.4.1. Experimental Research on Photoelectric Signal Measurement Method

**Figure 12:**
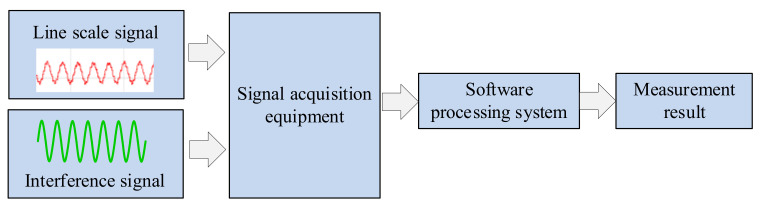
Data processing principle of single slit photoelectric signal measurement method.

The principle of the single slit photoelectric signal measurement method in this measurement system is shown in Figure 12. The line signal and laser interference signal were collected and stored in a computer, and the measurement results were obtained by processing the signal through a software system. Under the same measurement signal conditions, using software processing methods can improve measurement accuracy. As the input of the measurement system is a digital quantity, different measurement functions can be easily achieved through modular algorithms compared to conventional methods, thus possessing many advantages such as low cost, maintainability, observability, and reproducibility that conventional technical methods cannot achieve.

#### 3.4.2. Optical Image Data Processing Methods

**Figure 13:**
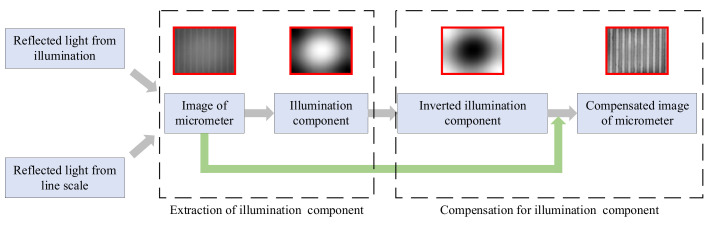
Schematic diagram of the principle of the line extraction method based on illumination compensation.

**Figure 14:**
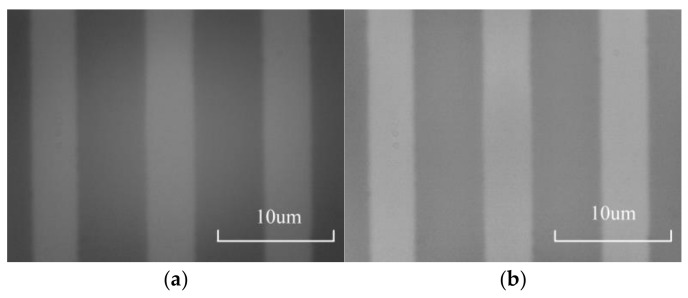
Image of the microscale after illumination compensation. (**a**) Original image. (**b**) Processed image.

**Figure 15:**
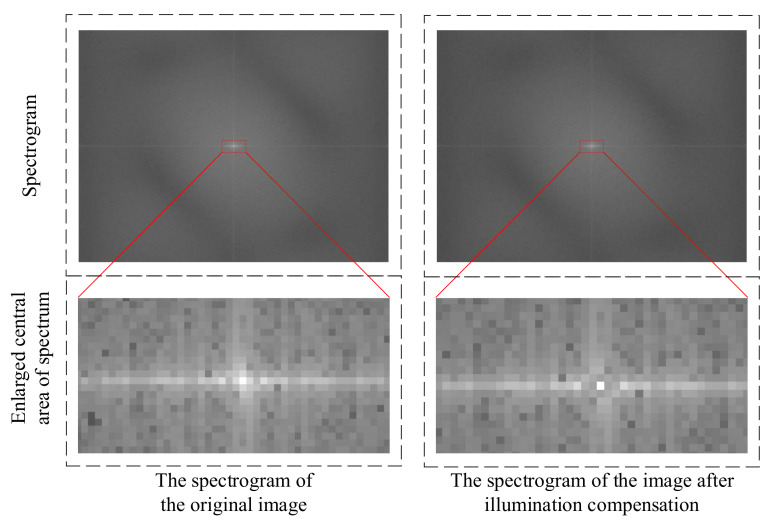
Spectrogram of the image before and after illumination compensation.

In image measurement methods, due to the scattering of light energy after magnification with the microscope, the overall grayscale of the microscopic image is low, and the contrast between the line image and the background is low. At the same time, due to the illumination of coaxial light sources, there are differences in brightness between the middle and surrounding areas of the image. It is necessary to preprocess the source image. This paper proposes a method for improving the uniformity of microscopic images based on illumination component extraction compensation, which is used for extracting line information in microscopic images. The image of a microscale should include two parts. The first part is the reflected light on the surface of the microscale, and the second part is the reflected light on the lines of the microscale. The first part reflects the lighting characteristics of the imaging system, usually low-frequency signals, while the second part contains the line information of the microscale, which includes high-frequency signals. It is very difficult to directly extract line information from the image, but the illumination component is relatively easy to process. Based on the above premise, this article proposes a method for improving the uniformity of microscopic images based on illumination component extraction compensation. The principle of this method is shown in Figure 13:

This method consists of two steps. Firstly, the illumination component is extracted, and the original image is converted into a grayscale image. As the illumination component is a low-frequency signal, low-pass filtering can be applied to the grayscale image to obtain the illumination component. After extraction, it can be found that the brightness of the middle area of the image is greater than that of the surrounding areas. The second step of the method is illumination compensation, which involves inverting the extracted illumination components. The inverted illumination components are weighted and overlaid with the original image to eliminate uneven illumination in the original image. The above method was used to process the image shown in Figure 14a. The processed image is shown in Figure 14b, and the spectrum of the image before and after processing is shown in Figure 15.

By observing the spectrogram, it can be seen that there is a large bright spot in the center area of the original image spectrogram, indicating a large amplitude of low-frequency signals in the image. After illumination compensation, there is only one pixel-sized bright spot in the center of the image spectrogram, indicating that the low-frequency component of the image is very small.

After image preprocessing, the edge and center of the line were recognized, and the image was filtered and enhanced, and other operations can be performed to obtain a good line image *F*. The extraction of the line center in this paper was based on a one-dimensional edge extraction algorithm, and the algorithm steps are as follows:

(1) Convert the line image *F* into a two-dimensional matrix *A*, assuming the image size to be *m* rows and *n* columns, and if *i* represents row *i* and *j* represents column *j*, then *F*(*i*,*j*) = *A*(*i*,*j*).

(2) Apply mean filtering to each row of matrix *A* to make the data smoother, and after mean filtering, obtain matrix *A*_M_.

(3) Starting from the first row of matrix *A*_M_, the peak position of each row is detected. Theoretically, the peak position is the center position of the line. However, due to the presence of interference information in the image, the peak position is prone to deviation. Therefore, this peak position is used for rough positioning of the line.

(4) Calculate the edge positions of the lines on both sides of the peak and determine 50% of the peak height as the edge of the line. Assuming there are a total of *k* lines, the edge of the line in the *i*-th row is represented as $e{\left(i\right)}_{11},e{\left(i\right)}_{12},e{\left(i\right)}_{21},e{\left(i\right)}_{22},\ldots ,e{\left(i\right)}_{k1},e{\left(i\right)}_{k2}$.

(5) Calculate the center position of the line; taking the midpoint of the two edges as the center position of the line, the center position of the *i*-th line is $e{\left(i\right)}_{1}=\frac{e{\left(i\right)}_{11}+e{\left(i\right)}_{12}}{2},e{\left(i\right)}_{2}=\frac{e{\left(i\right)}_{21}+e{\left(i\right)}_{22}}{2},\ldots ,e{\left(i\right)}_{k}=\frac{e{\left(i\right)}_{k1}+e{\left(i\right)}_{k2}}{2}$.

(6) Organize the line center position data to form a two-dimensional data array of *m* rows and *k* columns:

(7) $$C=\left[\begin{array}{cccc} e{\left(1\right)}_{1} & e{\left(1\right)}_{2} & \ldots & e{\left(1\right)}_{k}\\ e{\left(2\right)}_{1} & e{\left(2\right)}_{2} & \ldots & \ldots \\ \ldots & \ldots & \ldots & \ldots \\ e{\left(m\right)}_{1} & \ldots & \ldots & e{\left(m\right)}_{k} \end{array}\right]$$

(7) Taking each column of the two-dimensional data array *C* for regression analysis, the Equation for the *p*-th centerline can be obtained as follows [15]:

(8) $$y_{p}=\left(\frac{1}{m}\sum_{t=1}^{m}e{\left(t\right)}_{p}-\frac{m\sum_{t=1}^{m}t\sum_{t=1}^{m}te{\left(t\right)}_{p}-{\left(\sum_{t=1}^{m}t\right)}^{2}\sum_{t=1}^{m}e{\left(t\right)}_{p}}{m\sum_{t=1}^{m}t^{2}-{\left(\sum_{t=1}^{m}t\right)}^{2}}\right)+\frac{m\sum_{t=1}^{m}te{\left(t\right)}_{p}-\sum_{t=1}^{m}t\sum_{t=1}^{m}e{\left(t\right)}_{p}}{m\sum_{t=1}^{m}t^{2}-{\left(\sum_{t=1}^{m}t\right)}^{2}}x_{p}$$

(8) Calculate the distance between centerline Equations in pixels.

**Figure 16:**
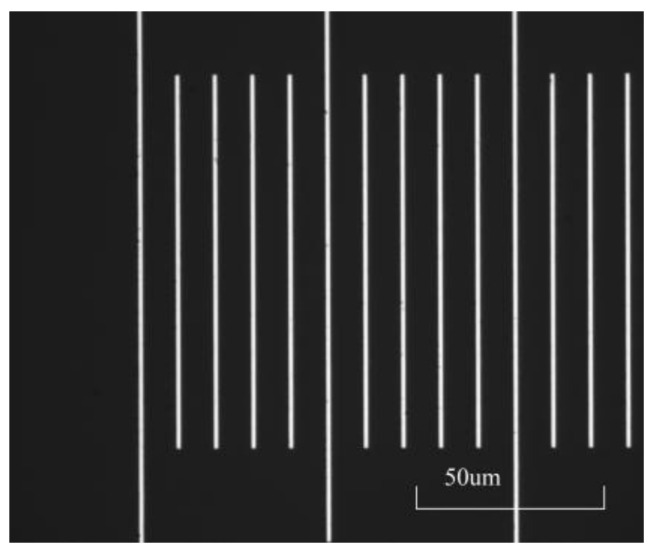
Microscale images used to verify the accuracy of the algorithm.

To verify the feasibility of the line center extraction algorithm based on one-dimensional edge extraction, a microscale image was captured using a primary system, and the image was processed using the image processing algorithm proposed in this paper. The line spacing was calculated and compared with the reference value of the primary system. The captured image is shown in Figure 16. After calculation, the processing results of the algorithm in this article are shown in Table 1 compared to the processing results of the primary system. The data in the table indicate that the maximum difference between the measurement results of this algorithm and the reference value was −0.017 μm. The results indicate that the above image processing program has universal applicability.

**Figure 17:**
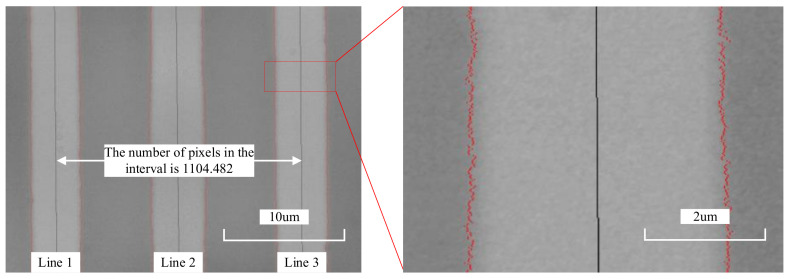
Line edge and center identification results.

The results of using this method to identify the edges and centers of the lines are shown in Figure 17. According to the calculation, the number of pixels between line 1 and line 3 in the image is 1104.482. In order to connect the image with the actual distance, it is necessary to calibrate the pixels. The pixel distance *D*_pixel_ has the following relationship with the actual distance *D*_actual_:

(9) $$D_{\mathrm{actual}}=\frac{D_{\mathrm{pixel}}\times s_{\mathrm{pixel}}}{\beta }$$

In the formula, *s*_pixel_ represents the pixel size and *β* represents the actual magnification of the imaging system; there is an error between the actual magnification of the imaging system and the nominal magnification. In actual calculations, the actual magnification should be used. Equation (9) is organized as follows:

(10) $$D_{\mathrm{actual}}=D_{\mathrm{pixel}}\times \frac{s_{\mathrm{pixel}}}{\beta }=k_{\mathrm{pixel}}\times D_{\mathrm{pixel}}$$

Among them, *k*_pixel_ is defined as the pixel equivalent.

The measuring range of the microscale shown in Figure 17 is 1 mm, and the spacing between the lines is 0.01 mm. It is calibrated according to the JJF1917-2021 [16] (Chinese standard) standard. The calibration spacing between the lines 1 and 3 in the image is 19.907 μm. According to the slope of the center of the lines in the image, it can be seen that the image has a tilt of 0.57°. Therefore, both horizontal and vertical pixels are calibrated. After calculation, the pixel equivalent for both horizontal and vertical pixels is 0.01802 μm.

Based on the above calibration results, for an image captured during the measurement process, we calculate the actual distance *D*_actual_ between the center of the line and the reference position, and *D*_actual_ is the measurement result of *d*_img_ in Equation (6).

## 4. Experiments and Uncertainty Evaluation

### 4.1. Experiment and Result Analysis

**Figure 18:**
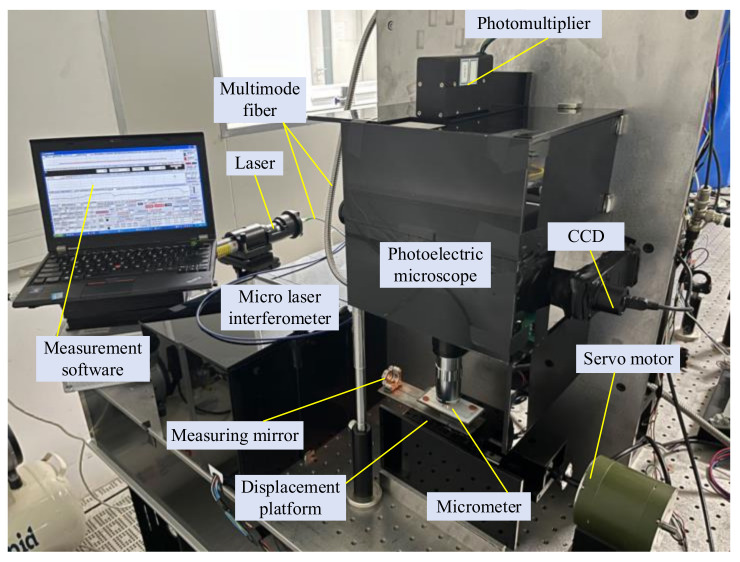
Microscale measurement system based on optoelectronics and image integration.

To verify the feasibility of the integrated measurement system, an experimental platform was built as shown in Figure 18. The microscale was measured using this platform, with an ambient temperature of (20 ± 0.2) °C and humidity of (50 ± 10)%. The measured values were compared with the reference values from the Chinese national primary standards of metrology.

#### 4.1.1. Experimental Analysis of Single Slit Optoelectronic Signal Measurement Method Combining Software and Hardware

**Figure 19:**
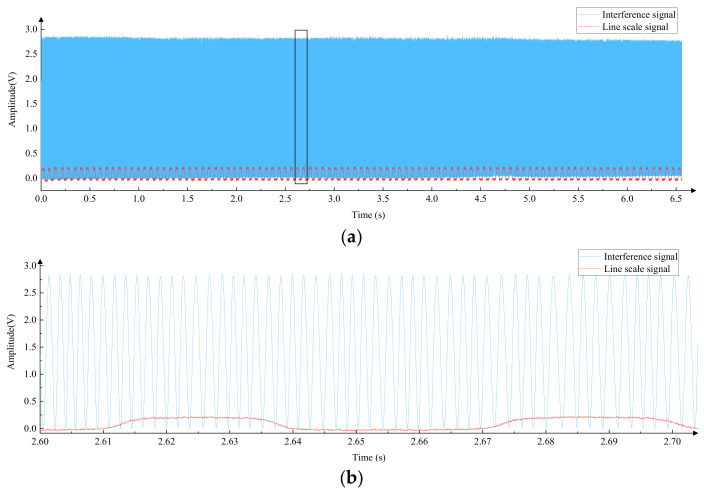
Microscale measurements of photoelectric scanning signals. (**a**) Single scan signal. (**b**) Localized magnification of single scan signal (The black box in Figure 19a).

The method was used to measure the microscale used for pixel equivalent calibration in Section 3.4, and line and interference signals were collected. The slit width used for measurement was 0.05 mm, and the actual width of the line was about 4 μm. A 150× objective was used, and the magnified image width of the line was about 0.6 mm. The width of the line image was much larger than the slit width, and the motion speed of the microscale was about 0.15 mm/s. Each measurement included forward and reverse measurements, with a total of three measurements taken. The average of the three measurements was taken as the final measurement result. During the measurement process, a scanning signal is shown in Figure 19. Due to the small width of the slit, the amplitude of the line signal is low, and the interference signal is a sine wave. The displacement platform has good stability in motion.

**Figure 20:**
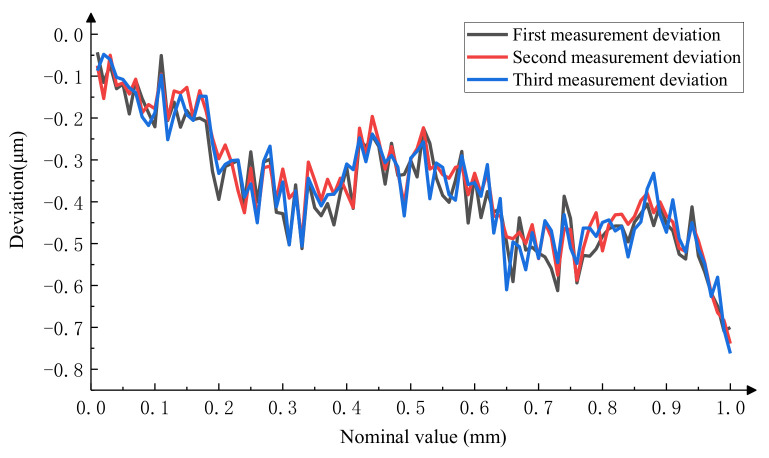
Results of three repetitions of the photoelectric signal method.

**Figure 21:**
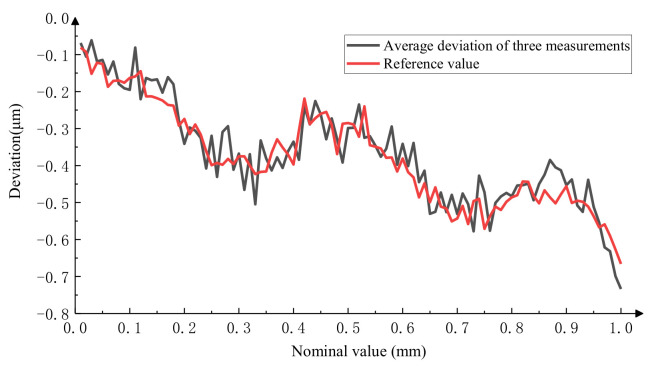
Comparison of the measurement results of the photoelectric signal method with standard values.

After calculation, the results of the three repeated measurements are shown in Figure 20, with a measurement repeatability of less than 0.078 μm. The relationship between the average of the three measurements and the reference value of the microscale is shown in Figure 21, which shows that the consistency between the measurement results of the integrated system and the reference value is better, with a maximum measurement difference of 0.105 μm.

#### 4.1.2. Experimental Analysis of Image Measurement Methods

**Figure 22:**
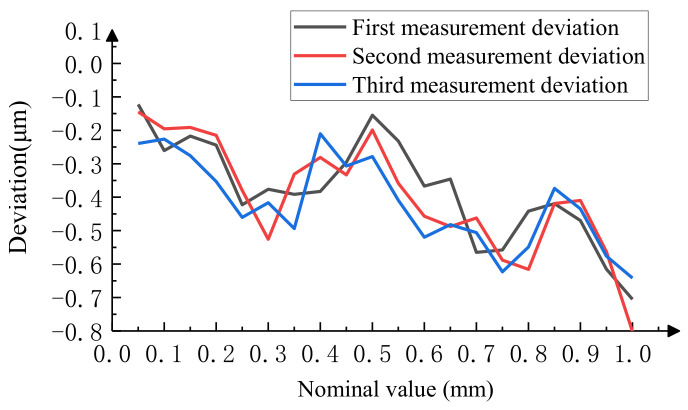
Results of three repeated measurements by image method.

**Figure 23:**
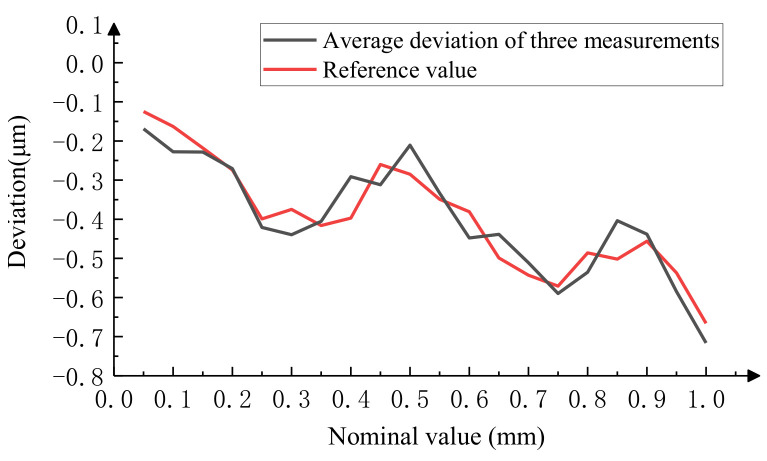
Comparison of image method measurement results with reference values.

Using an image-based method to measure the same microscale mentioned above, due to its slow measurement speed, 20 lines were measured at equal intervals for a total of three measurements. We took the average of three measurements as the final measurement result. The results of the three repeated measurements are shown in Figure 22, and the measurement repeatability was less than 0.105 μm; the final measurement result is shown in Table 2. Figure 23 intuitively shows the relationship between the final measurement result and the reference value of the microscopic scale. The data show that the measurement result of this method has good consistency with the reference value, with a maximum measurement difference of 0.106 μm.

**Figure 24:**
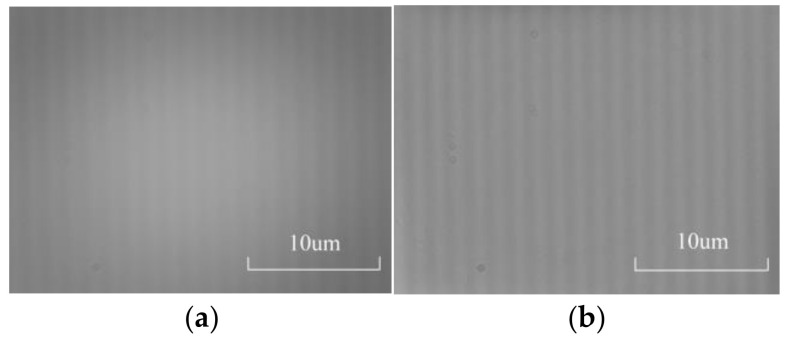
Microscale images with a line spacing of 1.5 μm captured in the experiment. (**a**) The microscale image with a 1.5 μm line spacing. (**b**) Microscale image processed using the method in this article.

As shown in Figure 24, a microscale image with a 1.5 μm line spacing was captured in the experiment. The photoelectric signal measurement method is no longer able to collect effective line signals, but the photoelectric image method can obtain effective marking information through image processing technology, indicating that the integrated measurement system can complete measurements that cannot be completed by a single photoelectric signal measurement method.

### 4.2. Uncertainty Assessment

Uncertainty is an important parameter that characterizes measurement accuracy and is also a component of measurement results [17]. This section lists the main components of uncertainty for different measurement methods, and quantifies and synthesizes them.

#### 4.2.1. Uncertainty Assessment of Single Slit Photoelectric Signal Measurement Methods

According to the measurement principle, the measurement model of the photoelectric signal measurement method is as follows:

(11) $$l=\frac{N\times \lambda_{0}}{2\times n(p_{\mathrm{air}},t_{\mathrm{air}},f_{\mathrm{air}})}+\alpha \times L_{0}\times (20-t_{s})+\delta l_{\mathrm{Abbe}}+\delta l_{\cos}$$

In the formula, *N* represents the counting of interference fringes, *λ*_0_ represents the vacuum wavelength of the laser, *α* represents the linear expansion coefficient of the material, *L*_0_ represents the length to be measured, *t*_s_ represents the material temperature, $\delta l_{\mathrm{Abbe}}$ represents Abbe error, $\delta l_{\cos}$ represents cosine error, *n*(*p*_air_, *t*_air_, *f*_air_) is the refractive index of air calculated using the Edlen formula (1998 version), *p*_air_ represents air pressure, *t*_air_ represents air temperature, and *f*_air_ represents air humidity. The specific expression of the Edlen formula is as follows [18]:

(12) $${\left(n-1\right)}_{s}\times 10^{8}=8091.37+\frac{2333983}{130-\sigma^{2}}+\frac{15518}{38.9-\sigma^{2}}$$

(13) $${\left(n-1\right)}_{x}=\left[1+0.5327\left(x-0.0004\right)\right]{\left(n-1\right)}_{s}$$

(14) $${\left(n-1\right)}_{pt}=\frac{p{\left(n-1\right)}_{s}}{93214.60}\times \frac{1+10^{-8}\left(0.5953-0.009876t\right)p}{1+0.0036610t}$$

(15) $$n_{ptf}-n_{pt}=-f\left(3.8020-0.0384\sigma^{2}\right)\times 10^{-10}$$

Equation (12) is the standard refractive index calculation formula under dry air and σ is the wave number in vacuum in μm^−1^; Equation (13) is the refractive index calculation formula for standard dry air at a CO_2_ content of *x*; Equation (14) is the refractive index calculation formula for standard dry air at a temperature of t and a pressure of *p*; and Equation (15) is the refractive index calculation formula for standard wet air at the water vapor pressure of *f*. The vacuum wavelength of the laser used in the experiment is 632.99 nm. After calculation, the effect of CO_2_ concentration on the refractive index of air can be ignored.

The parameters in the measurement model (11) are independent of each other; therefore, the evaluation model for measurement uncertainty is as follows:

(16) $$u_{c}=\sqrt{c_{s}^{2}u_{s}^{2}+c_{N}^{2}u_{N}^{2}+c_{\lambda 0}^{2}u_{\lambda 0}^{2}+c_{n}^{2}u_{n}^{2}+c_{\alpha }^{2}u_{\alpha }^{2}+c_{ts}^{2}u_{ts}^{2}+c_{\mathrm{abbe}}^{2}u_{\mathrm{abbe}}^{2}+c_{\cos}^{2}u_{\cos}^{2}}$$

In the formula, *u_i_* represents the uncertainty component and *c_i_* represents the sensitivity coefficient of the uncertainty component, which is obtained by calculating the partial derivative of the measurement model. *u_s_* represents the uncertainty component introduced by repeatability, and the other *u_i_* corresponds to various parameters in the measurement model. *u_N_* is combined from two uncertainty components, namely, the uncertainty component introduced by resolution and the uncertainty component introduced by nonlinearity. *u_n_* is combined from the three uncertainty components, namely, the uncertainty component introduced by inaccurate environmental temperature measurement, the uncertainty component introduced by inaccurate environmental humidity measurement, and the uncertainty component introduced by inaccurate environmental pressure measurement.

Based on the above model, we evaluate the uncertainty in measuring the 1 mm length of the microscale in Section 4.1.1. The components of the uncertainty are shown in Table 3. The letter “N” in the table indicates that the uncertainty component follows a normal distribution, while the letter “R” indicates that the uncertainty component follows a rectangular distribution

The uncertainty evaluation results indicate that the standard uncertainty of this method in measuring a 1 mm length is 0.068 μm. The normalization error is calculated using Equation (17) [19]:

(17) $$E_{n}=\frac{Y_{j}-Y_{r}}{k\cdot u}$$

In the formula, *Y_j_* represents the measurement result of this measurement system and *Y_r_* represents the reference value, which comes from the Chinese national primary standards of metrology. The measurement uncertainty of the reference value is *U* = 0.02 μm, *k* = 2, and *k* represents the inclusion factor, which is usually *k* = 2; *u* represents the standard uncertainty of *Y_j_* -*Y_r_*, and $u=\sqrt{u_{Yj}^{2}+u_{Yr}^{2}}$. After calculation, |*E_n_* | ≤ 1 and the measurement result is acceptable.

#### 4.2.2. Uncertainty Assessment of Image Measurement Methods

The measurement model of the image measurement method is as follows:

(18) $$l=\frac{N\times \lambda_{0}}{2\times n(p_{\mathrm{air}},t_{\mathrm{air}},f_{\mathrm{air}})}+\alpha \times L_{0}\times (20-t_{s})+d_{\mathrm{img}}+\delta l_{\mathrm{Abbe}}+\delta l_{\cos}$$

In the formula, $d_{\mathrm{img}}=\frac{D_{s}}{D_{\mathrm{pixel}1}}\times D_{\mathrm{pixel}2}$ *D*_s_ represents the reference value of the spacing between the lines used for calibration, *D*_pixel1_ represents the distance of the lines used for calibration in the image, and *D*_pixel2_ represents the distance of the measured lines in the image.

The parameters in the measurement model (18) are independent of each other; therefore, the evaluation model for measurement uncertainty is as follows:

(19) $$u_{c}=\sqrt{c_{s}^{2}u_{s}^{2}+c_{N}^{2}u_{N}^{2}+c_{\lambda 0}^{2}u_{\lambda 0}^{2}+c_{n}^{2}u_{n}^{2}+c_{\alpha }^{2}u_{\alpha }^{2}+c_{ts}^{2}u_{ts}^{2}+c_{\mathrm{dimg}}^{2}u_{\mathrm{dimg}}^{2}+c_{\mathrm{abbe}}^{2}u_{\mathrm{abbe}}^{2}+c_{\cos}^{2}u_{\cos}^{2}}$$

In the formula, *u*_dimg_ represents the uncertainty component introduced by inaccurate image processing, and *c*_dimg_ represents the sensitivity coefficient of this uncertainty component. According to the measurement model, most of the uncertainty components are listed in Table 3, and the description is not repeated in this section. The measurement uncertainty component introduced by the repeatability becomes 60 nm, and the image measurement method also adds the uncertainty component introduced by the inaccuracy of image processing. According to Section 3.4.2, the maximum difference between the measurement results of the image measurement method and the reference value was −0.017 μm. The reference value comes from the Chinese national primary standards of metrology, with an expanded uncertainty of *U* = 0.03 μm, *k* = 2. Assuming a uniform distribution, the uncertainty component introduced by an inaccurate image measurement was 0.012 μm. Once combined, the standard uncertainty of a 1 mm microscale measurement with the image measurement method was 0.080 μm. Taking the inclusion factor *k* = 2, the difference between the measurement result of the image measurement method and the reference value is in the allowable range of the uncertainty; the normalized error |*E_n_*| ≤ 1, and the result of the measurement can be accepted.

## 5. Conclusions and Prospect

In this paper, a high precision measurement system for microscales with photoelectric and image integration was investigated, and the system integrated the photoelectric signal measurement method and the image measurement method. The method of smooth motion based on ordinary linear guides was studied, and the laser interference signal shows that the smoothness of the motion was good. We analyzed the methods for reducing the cosine error under small-range conditions and studied the effects of mirror and optical axis deflection on the cosine error. We propose an image-based line positioning method that can accomplish measurement tasks that are difficult to achieve using optoelectronic signal measurement methods. Through the measurement experiments, it is verified that the measurement results of the system have consistency with the reference value, and the uncertainty evaluation results indicate that the standard uncertainty of the photoelectric signal measurement method is 0.068 μm when measuring a 1 mm length, the standard uncertainty of the image measurement method is 0.080 μm when measuring 1 mm length, and the normalized error of the measurement results of the two methods from the reference value is less than 1, which indicates that there is a good consistency between the measurement results and the reference value.

The measurement system studied in this article has the characteristics of being low cost, portable, and highly universal. The algorithm developed can process images from nonlocal systems and can handle different microscale images. It is relatively easy to transplant to fields such as image measurement and machine vision. The optoelectronic and image integration microscale measurement system can be expanded to two-dimensional measurements. With the in-depth study of image measurement methods, the system will be able to measure the size of more complex patterns and can meet the calibration needs of the many types of microscales. After calibrating the CCD pixel unit size, the system can also calibrate the magnification of the objective.

## Figures and Tables

**Table 1:** Comparison of the processing results of the line center extraction algorithm based on one-dimensional edge extraction with standard values.

Nominal Value (μm)	Standard Value (μm)	Result of the Algorithm in this Article (μm)	Difference from Standard Value (μm)
10	9.996	9.996	0.000
20	19.994	19.997	0.003
30	30.014	30.007	−0.007
40	40.012	40.002	−0.010
50	50.010	50.001	−0.009
60	60.009	60.003	−0.006
70	70.007	70.000	−0.007
80	80.010	79.999	−0.011
90	90.010	89.999	−0.011
100	100.012	99.999	−0.013
110	110.021	110.004	−0.017
120	120.018	120.008	−0.010
130	130.020	130.014	−0.006

**Table 2:** Results of microscale measurements by image measurement methods.

Nominal Value (mm)	*X*_laser_ (mm)	*d*_img_ (mm)	*X*	Deviation (μm)	Reference Deviation (μm)
0.00	−0.005746	0.001272	−0.004474	/	/
0.05	−0.052661	−0.001645	−0.054306	−0.169	−0.125
0.10	−0.102523	−0.001724	−0.104247	−0.227	−0.163
0.15	−0.156075	0.001829	−0.154246	−0.228	−0.218
0.20	−0.206166	0.001962	−0.204204	−0.271	−0.274
0.25	−0.253046	−0.001008	−0.254054	−0.420	−0.399
0.30	−0.305140	0.001105	−0.304035	−0.439	−0.375
0.35	−0.352868	−0.001201	−0.354069	−0.406	−0.416
0.40	−0.405807	0.001624	−0.404183	−0.291	−0.397
0.45	−0.456289	0.002126	−0.454163	−0.312	−0.26
0.50	−0.505402	0.001138	−0.504264	−0.211	−0.285
0.55	−0.552320	−0.001821	−0.554141	−0.333	−0.349
0.60	−0.606117	0.002090	−0.604027	−0.448	−0.381
0.65	−0.655845	0.001809	−0.654036	−0.439	−0.499
0.70	−0.706136	0.002172	−0.703964	−0.511	−0.543
0.75	−0.755613	0.001728	−0.753885	−0.590	−0.571
0.80	−0.805841	0.001901	−0.803940	−0.535	−0.486
0.85	−0.855489	0.001418	−0.854071	−0.404	−0.502
0.90	−0.904973	0.000937	−0.904036	−0.438	−0.456
0.95	−0.952593	−0.001297	−0.953890	−0.584	−0.537
1.00	−1.005425	0.001667	−1.003758	−0.716	−0.666

**Table 3:** Measurement uncertainty components of photoelectric signal measurement methods.

Error Sources	$x_{i}$	$u(x_{i})$	Prob.	$c_{i}=\delta l/\delta x_{i}$	Unit	$u_{i}(l)$
Repeatability	*S*	45 nm	N	1.0	/	45 nm
Resolution	*N*_1_	0.58 nm	R	1.0	/	0.58 nm
Nonlinear	*N*_2_	21 nm	R	1.0	/	21 nm
Laser wavelength	$\lambda_{0}$	1.0 × 10^−7^ × $\lambda_{0}$	R	*L*/$\lambda_{0}$	/	1.0 × 10^−7^ *L*
Edlen formula	n	1.0 × 10^−8^	R	1.0	*L*	(1.0 × 10^−8^) × *L*
Air pressure	*p*_air_	10 Pa	R	2.70 × 10^−9^	*L*/Pa	(2.7 × 10^−9^) × *L*
Air temperature	*t*_air_	0.5 °C	R	9.23 × 10^−7^	*L*/°C	(0.5 × 10^−6^) × *L*
Air humidity	*f*_air_	30 Pa	R	3.67 × 10^−10^	*L*/Pa	(11 × 10^−9^) × *L*
Thermal linear expansion coefficient	α	8.0 × 10^−6^ °C^−1^	R	0.5	*L* °C	(4.0 × 10^−6^) × *L*
Material temperature	*t*_s_	0.5 °C	R	8 × 10^−6^	*L*/°C	(8 × 10^−6^) × *L*
Abbe error	$\delta l_{\mathrm{Abbe}}$	45 nm	R	1.0	/	45 nm
Cosine error	$\delta l_{\cos}$	3.2 × 10^−8^ nm	R	1.0	/	3.2 × 10^−8^ × *L*
Standard uncertainty (when *L*_0_ is 1 mm)						0.068 μm

## Data Availability

Data are available from the authors on request.
